# Germinated Pigeon Pea (*Cajanus cajan*): a novel diet for lowering oxidative stress and hyperglycemia

**DOI:** 10.1002/fsn3.343

**Published:** 2016-01-20

**Authors:** Nneka N. Uchegbu, Charles N. Ishiwu

**Affiliations:** ^1^ Department of Food TechnologyInstitute of Management and TechnologyEnuguNigeria; ^2^ Department of Food Science and TechnologyNnamdi Azikiwe UniversityAwkaNigeria

**Keywords:** Antioxidants, diabetes, phenolics, pigeon pea

## Abstract

This work studied the antioxidant activity of extract of germinated pigeon pea (*Cajanus cajan*) in alloxan‐induced diabetic rats. Germination was carried out in a dark chamber under room temperature (28°C). The total phenolic, 1,1,diphenyl‐2‐picrylhy‐drazyl free radical (DPPH) scavenging, the inhibition of *α*‐amylase and *α*‐glucosidase were done in vitro and blood glucose levels of the animal were investigated. Lipid peroxidation (LPO) and reduced glutathione (GSH) were analyzed spectrophotometrically. The total phenolic and DPPH scavenging activity increased by 30% and 63%, respectively, after germinating pigeon pea. Also after germination there was an increase in the inhibitory potential of pigeon pea extract against *α*‐glucosidase compared with the nongerminated pigeon pea extract. There was a significant increase (*P *<* *0.05) in fasting blood glucose level of alloxan‐induced rats. Consumption of germinated pigeon pea extract gave rise to a reduced fasting blood glucose level in diabetic rats. On administration of germinated pigeon pea extract, LPO reduced drastically but there was an increase in the level of GSH. This study concluded that intake of germinated pigeon pea is a good dietary supplement for controlling hyperglycemia and LPO.

## Introduction

Different people of all sexes and ages are being affected in one way or the other by diabetes mellitus (Marrazzo et al. 2014). It is a disorder associated with insufficient production of endogenous insulins and manifestation of hyperglycemia in the human body (Saravanana and Ponnusamy 2011). Maritim et al. (2003) reported that oxidative stress contributes immensely in development of diabetes (Palanisamy et al. 2011). This increased oxidative stress is accompanied by a decreased antioxidant capacity.

During the development of Type 2 diabetics mellitus, abnormalities in lipid and carbohydrate metabolism lead to increased oxidative stress which gives rise to high blood sugar level (Palanisamy et al. 2011). Breaking down of starch by pancreatic *α*‐amylase and absorption of glucose by intestinal *α*‐glucosidase lead to risen blood glucose level that causes Type 2 diabetes. Increase in lipids or lipoprotein leads to hyperlipidemia and could lead to oxidative stress (Rotimi et al. 2013). It has been documented that hyperlipidemia and hyperglycemia exist together in people suffering from diabetes (Rotimi et al. 2013).

Oxidative damages in people suffering from diabetes can be reduced by intake of antioxidants (Edziri 2012), but butylated hydroxyanisol (BHA) and butylated hydroxytoluene (BHT) could have adverse side effect in human because they are synthetic compounds.

Since most people are skeptical about the likely adverse effects of prolonged consumption of synthetic antioxidants, natural antioxidant of plant origin is becoming very popular (El‐Mawla and Husam 2011). There is a strong evidence that crops with antioxidant activity have the ability of exerting protective effect against oxidative stress in human (El‐Mawla and Husam 2011).

Legumes are rich source of bioactive compounds with antioxidant potentials. Pigeon pea is an excellent sources of polyphenolic compounds. It possess potent antihyperglycemic activity Ashok et al.(2013) and it is used in Panamanian folk medicine for the treatment of diabetes (Dolui and Sengupta 2012). Germinated pigeon pea exhibits more secondary metabolites than the ungerminated counterpart. During the first phase of germination, enzymes of biosynthetic nature are activated and thus, germination leads to high content of bioactive compounds with improved nutritional quality (El‐Adawy et al. 2003).

In this work, antioxidant, free radical scavenging, Type 2 diabetes relevant enzyme inhibitory potential, blood glucose concentration, lipid peroxidation (LPO), and nonprotein sulfhydryl group in the liver tissues of diabetic rats fed the germinated pigeon pea meal were investigated.

## Materials and Methods

Dried pigeon pea (*Cajanus cajan*) were purchased from Ogbete main market in Enugu state–Nigeria. The pigeon pea seeds were placed inside a sealed plastic container and kept in a refrigerator at 5°C before germination.

### Germinated process

The pigeon pea was soaked in 250 mL of water with 0.7% sodium hypochlorite for 30 min under atmospheric temperature of 28°C. The water was drained off. The seeds were soaked again in water for 5 h. The water was drained off and the seed placed on muslin cloth and allowed to germinate for 3 days at 28°C in the dark. (Lopez‐Amoros et al. 2006). The sprouted seeds were oven dried at 60°C for 4 h, Later they were ground and sieved with a sieve mesh size 0.18 mm into flour, which was then packaged. The nonsprouted seeds were ground, sieved, and packaged to serve as control.

### Extraction

A 200 g of both the sprouted and nonsprouted flour sample were extracted separately with the method of Siddhuraju (2007). The samples were stirred with 100 mL of 70% acetone for 24 h at 25°C and then filtered with Whatman No. 4 filter paper. The residues were further defatted with extract 50 mL of 70% acetone and stirred for 3 h. Rotary vacuum evaporator (RE 300, Xamato, Tokyo, Japan) was used to evaporate the solvent under reduced pressure at 40°C. The lypophilization (4KB TXL–75; Virtis Benchtop K, New York) was used to remove the remaining water. The dry powder was kept in an airtight plastic container at 0°C until needed.

### Determination of total phenolic content

The method of Makkar et al.(1993) was used to determine the total phenolic content of the sample. Sample extract (50 *μ*L) was put in a test tube and distilled water was used to make up the volume to 500 *μ*L. 250 *μ*L of folin‐Ciocalteu reagent was put inside the test tube and 1.25 mL of 20% sodium carbonate solution were also added to the test tube. The test tube was vortexed before incubating for 40 min in the dark. Absorbance was read at 725 nm, using spectrophotometer (Shimadzu, Australia).

### Determination of DPPH

The method of Blosis (1958) was used to determine the DPPH. 250 *μ*g/mL of pigeon pea extracted with acetone was dissolved in dimethyl sulfoxide inside a test tube. This was done in triplicates. Five microliters of 0.1 mol/L methanol solution of DPPH was then put into different test tubes and vortexed. It was allowed to rest for 20 min at 35°C. The control sample was prepared without adding the extracts. The absorbance (OD) of the sample was noted at 517 nm, using methanol for base line corrections (blank). The DPPH radical scavenging activity was expressed as 1% scavenging activity. The formula used in calculating radical scavenging activity is shown below.

$$\text{Radical scavenging activity}\;\%=\frac{\text{OD Control}-\text{OD Samples}}{\text{OD Control}}\times 100.$$

### Determination of *α*‐amylase and *α*‐glucosidase inhibitory potential

*α*‐amylase and *α*‐glucosidase inhibitory potential of germinated pigeon pea extracts were assayed with the method described by Abdulkadir et al. (2011). The *α*‐amylase and *α*‐glucosidase inhibitory activity was expressed as percentage inhibition and the formula used for the calculation is shown below.

$$\%\text{inhibition}=\frac{\Delta A\text{Control}-\Delta A\text{Extract}}{\Delta A\text{Control}}\times 100.$$

### Animal and diet

Adult Wistar‐Albino rats weighing between 150 and 200 g used for the experiments were brought from the Department of Animal Science, University of Nigeria, Nsukka–Nigeria. The rats were fed with normal rat diet for a period of 1 week in their individual metabolic cages, and were denied of diet, but were given water 16 h prior to the commencement of the experiments (Rotimi et al. 2013).

### Induction of diabetes

A single intarperitonial injection of alloxan monohydrate at a dose of 180 mg/kg body weight. dissolved in normal saline (2%) was used to induce diabetes mellitus. Only normal saline was given to rats in the control group. The fasting blood glucose of the rat was recorded after 7 days. Only those rats having fasting blood glucose level more than 220 mg/dL were used for the experiment.

### Experimental design

The rats were divided into three groups. Each contained six rats:

Group 1: Nondiabetic rats (control). The rats were given normal diet.

Group 2: Diabetic rats fed high fatty diet.

Group 3: Diabetic rats fed high fatty diet plus germinated pigeon pea meal.

After 4 weeks of treatment, the blood was collected and the animal decapitated. The experiment was conducted following regulation of the body governing animal experiment.

### Estimation of blood glucose levels

Glucometer‐elite commercial test (Bayer) was used to determine the blood glucose level. This was based on glucose oxidase method. Through the tip of the tail, the blood samples were collected. This collection was done after fasting the animal for 16 h, but they were allowed access to water. Blood sugar levels were determined at weekly interval for 4 weeks.

### Biochemical analysis

Lipid peroxidation and nonprotein sulfhydryl group (cellular reduced glutathione [GSH]) in liver screening was done, using standard procedure as described by Sendogdu et al. (2006) and Deliorman (2003), respectively.

### Statistical analysis

Results obtained were expressed as mean ± standard deviation. The mean was compared using one way analysis of variance. Data were considered not significant at *P* > 0.05 using the Ducan Multiple Range Test with SPSS software (version 17.0, SPSS Inc. Illinois, USA).

## Results and Discussion

Table 1 shows the content of total phenolic content. After germination of pigeon pea, total phenolic content increased by 30.12% (from 73.02 ± 0.02 to 95.01 ± 0.02 mg GAE/100 g dry weight. This increase in the amount of phenolic compound after germination is in accordance with the report of Lopez‐Amoros et al. (2006) which indicated that germination increased the quantity and quality of phenolic compounds in legumes. Another study that supported this finding was Duenas et al. (2009) who germinated lupin seeds (*Lupinus angustifolius L*.) and observed 46% increase in total phenolics.

**Table 1:** Total phenolic content (TPC) and DPPH in the samples

Sample	TPC, mg/GAE/100 g dry weight	DPPH, % scavenging
Germinated pigeon pea	95.01 ± 0.02^a^	85.2 ± 0.02^a^
Nongerminated pigeon pea	73.02 ± 0.02^b^	52.1 ± 0.04^b^

The values are expressed as mean ± SD.

Data in the same column bearing different superscripts are significantly different (*P *>* *0.05).

### Total phenolic content

Phenolic compounds exhibit high antioxidative activity, therefore they can be classified as antioxidant compounds. They act as free radical scavengers and terminators (Pourmorad et al. 2006) and play medicinal role in human body.

### DPPH scavenging activity

Various diseases such as cancer diabetes and central nervous system disorders could be caused by free radicals in the body. The scavenging ability of antioxidants is employed in the management of these degenerative diseases. DPPH stable free radical method is a more efficient and speedy way to assay the antioxidative capacity of a plant extracts (Pourmorad et al. 2006). Results showed 63.52% increase in the % scavenging of pigeon pea extract after germination. This result indicated that the germinated pigeon extract which contains higher amount of phenolic compound than the ungerminated one could exhibit higher antioxidant activity. This high scavenging ability of germinated pigeon pea extract might be due to the presence of hydroxyl group in the phenolic chemical structure. This hydroxyl group can provide the required component as a free radical scavenger. Biological reactions often produce free radical as by‐products. (Abheri et al. 2010) had reported the involvement of free radical in many pathogenesis. A potential scavenger of free radicals can lead to prevention of many diseases. Germinated pigeon pea is therefore a potential scavenger of free radicals.

### *α*‐glucosidase and *α*‐amylase inhibitory activity

Control of Type 2 diabetes through dietary practices is becoming popular in nutritional research. Oxidative stress due to hyperglycemia and hyperlipidemia can also be linked to diabetes.

**Figure 1:**
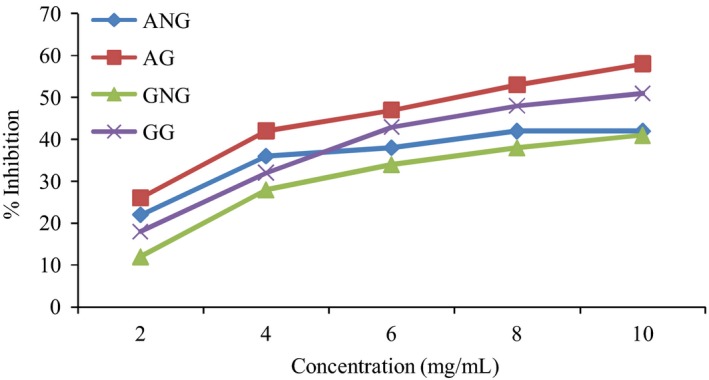
*α*‐amylase and *α*‐glucosidase inhibitory potential of germinated and nongerminated pigeon pea seed.

Due to the fact that Type 2 diabetes is associated with diet, intake of food with high content of *α*‐glucosidase and *α*‐amylase inhibitors could possibly be a novel method of treatment of this disease. Researchers now focus their interest on plant with medicinal potentials and functional foods in preventing and curing Type 2 diabetes. Due to side effect of synthetic inhibitors, plants containing these enzymes with antioxidant activity for managing patients suffering from Type 2 diabetes are being investigated (Vellingiri 2011). This study has shown that germinated pigeon pea exhibits significantly higher (*P* < 0.05) inhibitory activity against *α*‐amylase and *α*‐glucosidase than the nongerminated pigeon pea (Fig. 1). The estimated EC_50_ value of nongerminated and germinated pigeon pea for *α*‐glucosidase inhibition were 3.31 and 2.52 mg/mL, for *α*‐amylase inhibition. EC_50_ values of nongerminated and germinated were 2.95 and 2.41, respectively.

The results suggest that the enzyme (*α*‐glucosidase and *α*‐amylase) inhibitory potential of germinated pigeon pea has been correlated with the amount of phenolic content and antioxidant activity indicating the relevance of the increase in the therapeutic properties of germinated pigeon pea (Upadhyaya et al. 2011). *α*‐amylase catalyzes the breakdown of glycosidic linkages in the carbohydrate and gives away the broken products. The enzymatic degradation of the polymer constitutes the first step. Starch blockers, (*α*‐amylase inhibitors) bind with the reactive sites by altering its catalytic activity, thereby causing a decrease in blood glucose level, whereas the *α*‐glucosidase which is released from the cells lining of the small intestine results in the cleavage of di and oligosarccharide to glucose and its assimilation in the intestine.

This delay in the absorption of glucose can have a beneficial effect in the management of diabetes. *α*‐glucosidase inhibitor can slow down the rate at which glucose is absorbed in the intestine, by stopping the bondage of di and oligosaccharides through reversible inhibition of intestinal *α*‐glucosidase (Vellingiri 2011).

To this effect, the *α*‐glucosidase and *α*‐amylase inhibition activity noticed in germinated pigeon pea is recommended as the dietary management of Type 2 diabetes.

**Figure 2:**
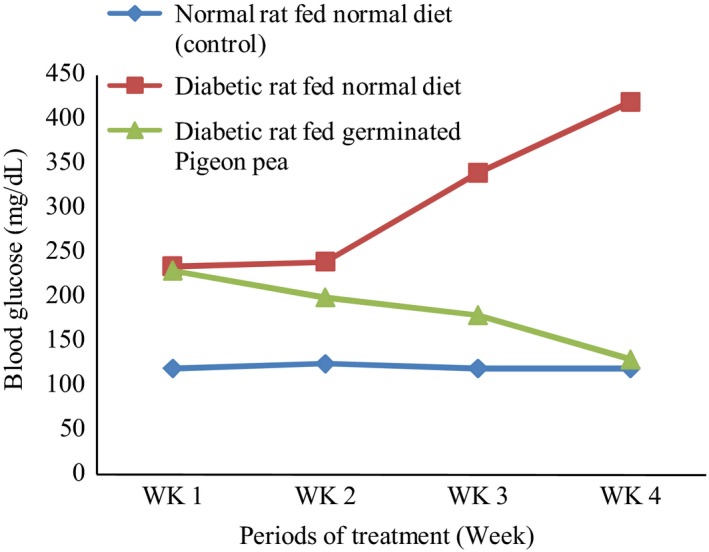
Blood glucose concentration of normal and diabetic rats fed normal, fatty, and germinated pigeon pea diets for 4 weeks.

Figure 2 shows the different levels of blood sugar in normal (control), diabetic control, and germinated pigeon pea extract – treated rat during 4‐week period of study. The blood glucose level of the control group was significantly lower than those of diabetic rats (*P* < 0.05). After feeding the rats with germinated pigeon pea meal, there was a significant reduction in the blood glucose level than that of the diabetic group that were not fed the germinated pigeon pea diet (*P* < 0.05). It has been established that raising blood glucose in diabetes could deplete cells of their antioxidant status with increase in free radical, thereby sparking off oxidative stress. The germinated pigeon pea with hypoglycemia potential may indirectly decrease the rate at which oxidative stress occurs by reducing the blood glucose level and behaving as free radical scavenging directly.

Table 2 shows the values of antioxidant activity. LPO and GSH serves as markers in studying the antioxidant activity. A significant elevation (*P* < 0.05) in the level of malondialdehyde (MDA) is noticed in liver tissue of hyperglycemia rats.

**Table 2:** Different levels of blood sugar in normal (control), diabetic control, and germinated pigeon pea extract–treated rat during 4‐week period of study

Groups	Control rat	Diabetic rat fed high fatty meal	Diabetic rat fed germinated pigeon pea meal
Lipid peroxidation, nmol MDA release/mg protein	1.31 ± 0.22^b^	2.01 ± 0.25^a^	1.43 ± 0.33^b^
GSH, *μ*g reduced GSH utilized/mm/mg protein	30.2 ± 1.7^b^	15.4 ± 2.1^a^	28.3 ± 1.7^b^

Values are mean ± SD.

Data on the same row bearing different superscript differed significantly (*P *<* *0.05).

MDA, malondialdehyde; GSH, reduced glutathione.

The germinated pigeon pea meal given orally resulted to a significant decrease in LPO in the liver (31.1%). A reduction in the concentration of GSH in the liver tissue of hyperlipidemia diabetic rat was also observed (40.6%). But, the administration of germinated pigeon pea meal significantly increased the level of GSH.

There is likelihood that animals which are both hyperglycemia could develop oxidative stress. LPO and GSH are used to mark oxidative stress. Increased LPO which causes oxidative stress can lead to several pathologies like inflammation, diabetes, aging, and renal failure (Karuna et al. 2009). The diabetic‐induced‐high fat fed rats showed increase LPO than nondiabetic (control) rats. The rats fed with germinated pigeon pea meal showed more reduced LPO than diabetic‐induced rats fed only with high fat food. GSH protects the cell constituents from oxidative damage (Rajasekaran and Kalaivani 2011). Decrease in glutathione is caused by oxidative stress. Concentration of the marker was found to be higher in rats fed with germinated pigeon pea meal than in those fed with nongerminated meal.

## Conclusion

The free radical scavenging activity of germinated pigeon pea meal in diabetic – associated hyperlipidemia has been demonstrated in this study. This may be associated with high content of total phenolics, increased antioxidant potential, and inhibitory potential of carbohydrate‐digesting enzymes. Therefore, this study concludes that intake of germinated pigeon pea is a good dietary supplement for controlling hyperglycemia.

## Conflicts of Interest

Conflicts of interest was not reported by the authors.
